# Supplementary material for: Comparative qualitative phosphoproteomics analysis identifies shared phosphorylation motifs and associated biological processes in evolutionary divergent plants
Source: J Proteomics. 2018 Jun 15;181:152–9. doi: 10.1016/j.jprot.2018.04.011 (PMC5971217; doi:10.1016/j.jprot.2018.04.011)
Supplement: Supplementary Fig. 1 — The length distribution of phosphorylated, non-phosphorylated and all peptides in rice and Arabidopsis. [file mmc1.pdf]

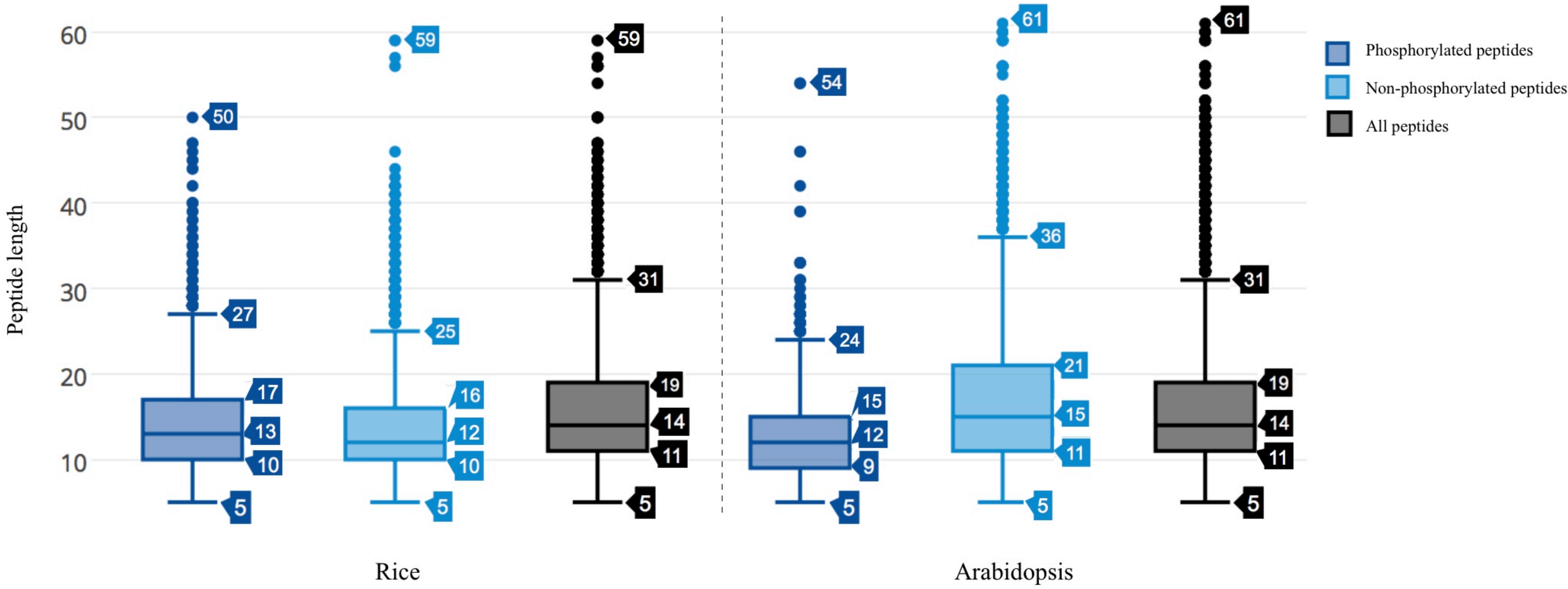


Supplementary Figure 1. The length distribution of phosphorylated, non-phosphorylated and all peptides in rice and *Arabidopsis.*
